# Cancer du sein historique à propos d'un cas!!! Comment réagir

**DOI:** 10.11604/pamj.2016.24.56.8721

**Published:** 2016-05-13

**Authors:** Omar Laghzaoui

**Affiliations:** 1 Laghzaoui, Service de Gynécologie Obstétrique de l'Hôpital Militaire Moulay Ismail, Université Sidi Mohammed Ben Abdellah, Faculté de Médecine et de Pharmacie, Fès, Maroc

**Keywords:** cancer du sein, retard diagnostic, étiologies, sensibilisation, information, Breast cancer, delayed diagnosis, etiologies, awareness, information

## Abstract

Le cancer du sein localement avancé est une entité qui se fait rare dans les pays développés alors qu'on continue de recevoir des patientes à un stade avancé dans les pays africains. Nous proposant le cas d'une patiente intellectuelle prise en charge à l'hôpital militaire, Meknès, Maroc; qui a décelé la présence d'un nodule du sein droit par l'autopalpation mais elle n'a consulté le médecin que six mois après, dans un état historique de cancer du sein localement avancé. Le but de notre publication est donc de soulever les anomalies contemporaines aboutissant au retard diagnostic du cancer du sein et de proposer des solutions pour mieux sensibiliser la population.

## Abstract (en)

Locally advanced breast cancer is a rare entity in developed countries while we continue to treat patients with advanced stage tumor in African countries. Our study reports the case of an intellectual patient referring the presence of a right breast nodule found during self-examination treated at the military hospital of Meknes, Morocco; she was examined only six months after discovery, in a historical state of locally advanced breast cancer. Our study aims to detect the contemporary anomalies leading to a delayed diagnosis of the breast cancer and to propose solutions to increase awareness among population.

## Introduction

Le cancer du sein localement avancé est une hantise dans notre pratique quotidienne. Les étiologies qui poussent les femmes à consulter tardivement sont généralement en rapport avec la pauvreté intellectuelle et économique, mais de nos jours on assiste à une novelle cause du retard diagnostic émanant de la mauvaise compréhension des sujets médicaux traités sur le net.

## Patient et observation

**Figure 1:**
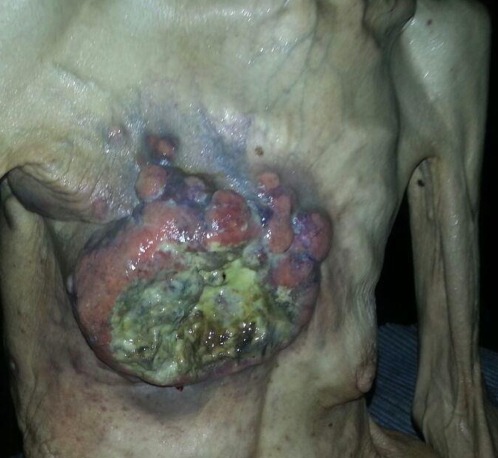
Cancer du sein localement avancé (historique) chez une patiente de 45 ans

Patiente âgée de 45 ans bibliothécaire de fonction, adepte à l'automédication orientée par certains sites internet. Elle est multipare ayant allaitée cinq enfants, non fumeuse, sans notion de cancer dans la famille et n'ayant jamais utilisée de contraception hormonale. Elle rapporte l'apparition six mois auparavant d'un nodule de petite taille au niveau du quadrant supero-externe du sein droit douloureux à l'autopalpation dans un contexte d'amaigrissement qui est mis sur le compte d'un auto-régime hypocalorique entrepris par la patiente à cause de son surpoids. La patiente a faussement éliminé le diagnostic de cancer du sein en se basant sur l'absence des facteurs de risque et la douleur du nodule. L'apparition des ulcérations de la peau faisant penser la patiente au zona et traité entant que tel ce qui a retardé d'avantage le diagnostic. L’évolution de la lésion s'est faite d'une façon spectaculaire et rapide dans le temps vers l'extension à la peau avec un amaigrissement cadavérique ce qui a motivé une consultation au service de gynécologie de l'hôpital militaire de la ville de Meknès. L'examen à l'admission objective une patiente d'un teint cireux, cachectique, déshydraté dont l’état général est altéré; pesant 40 kilogrammes avec un amaigrissement chiffré à 36 kilogrammes en six mois. L'examen mammaire révèle un ensemble de lésions nodulaires du sein droit, confluentes ulcérées par endroit, occupant presque la totalité de l'hémi-thorax, auquel elle adhère intimement (Figure 1). L'aire axillaire homolatérale est comblée par un paquet ganglionnaire de six centimètre de grand axe. Le sein controlatéral ne présente pas de lésion, la palpation abdominale met en évidence une hépatomégalie nodulaire. Une macro-biopsie du sein réalisée objective un carcinome canalaire infiltrant, grade III SBR avec emboles vasculaires; les récepteurs hormonaux sont positifs aussi bien pour les œstrogènes que la progestérone et une surexpression du HER2. Le bilan d'extension découvre une métastase osseuse au niveau du col fémorale droit et plusieurs nodules métastatiques hépatiques. Un traitement palliatif à base de radio-chimiothérapie plus le trastuzumab est décidé par le staff multidisciplinaire d'oncologie de l'hôpital, mais malheureusement la patiente est décédée juste après la deuxième cure de chimiothérapie dans un tableau de défaillance multi viscérale.

## Discussion

Le cancer du sein vient au premier rang des cancers de la femme au Maroc. Selon le registre des cancers de la ville de Rabat son Incidence est de 20,5 pour 100 000 habitants et il est responsable de 300 000 décès annuel, en partie en rapport avec un diagnostic tardif. 63,07% des patientes consultent à un stade localement avancé et 13,84% aux stades de métastases [1]. Ce retard diagnostic est en rapport selon la littérature avec l'ignorance, la pauvreté et les habitudes socioculturelles auxquelles s'ajoute ces dernières années l'auto-prise en charge médicale basée sur l'information apportée par le net [1–3]. On soulève ainsi d'après une étude en 2012 du Haut-Commissariat au plan, que près de 30% des Marocaines de 15 ans et plus sont analphabètes et donc incapables d'interagir avec toutes sortes d'informations médicale. C'est une tranche de population qui ne peut émerger que par des programmes gouvernementaux et communautaires pour lutter contre l'analphabétisme ainsi que l’élaboration d'une information audio-visuelle simple à assimiler quant à la nécessité du dépistage précoce du cancer du sein [1, 3–5]. L'absence d'une carte sanitaire au Maroc est responsable d'une disparité de répartition des formations médicales avec une concentration des hôpitaux et des praticiens dans les grandes villes laissant un vide ailleurs. L'accès géographique au dépistage du cancer du sein se trouve ainsi compromis pour une partie de la population. La solution réside dans le rapprochement des structures sanitaires des citoyens avec la création d'une carte sanitaire faisant profiter toute la population des soins médicaux [1, 4–8]. Le manque de moyens financiers et l'absence de couverture sociale est un élément capitale dans le retard diagnostic du cancer du sein, une mammographie coûte près de 50 euros ce qui représente le quart du Salaire minimum interprofessionnel garanti au Maroc (SMIG en 2014 est 209 euros). Le Maroc vient de se doter depuis 2012 d'un système de couverture médicale gratuite appelé RAMED (Régime d'Assistance Médicale aux populations démunis) mais il n'intéresse que 10% de la population, une généralisation de la couverture sociale pourrait contribuer au diagnostic précoce du cancer du sein [1]. Le traitement traditionnel est ancré dans les habitudes socioculturelles marocaines ainsi que d'autres pays africains et asiatiques. Ce traitement passe en premier plan dans un groupe de population particulière causant un retard diagnostic du cancer du sein et même une source de complications infectieuses voire toxique [1, 3]. Au Maroc on compte 56% d'internautes en 2013, ce qui parait bien comme outil d'information, mais l'interprétation ambiguë des informations médicales peut être néfaste et causer un retard diagnostic. C'est le cas de notre patiente qui a substitué l'internet au médecin pour écarter l’éventualité du cancer du sein en interprétant mal la notion d'absence des facteurs de risque du cancer du sein. La mondialisation de l'information médicale à travers internet est une arme à double tranchant, elle apporte certes un éclaircissement important dans le domaine médical mais peut entraîner une confusion si l'utilisateur n'a pas le niveau intellectuel nécessaire pour bien assimiler l'information. Cette situation pose un défi aux responsables de la santé publique qui devraient éveiller toutes les classes sociale pour mieux utiliser le net [9, 10].

## Conclusion

Le retard diagnostic du cancer du sein au Maroc est lié plusieurs facteurs entremêlés on en connaissait trois, l'ignorance, la pauvreté, et les habitudes socio-culturelles auxquels s'ajoute actuellement la mauvaise interprétation des informations médicales du net. La précocité du diagnostic du cancer du sein passe donc par l’éducation, la généralisation de la couverture sociale médicale, la lutte contre la pauvreté et les traitements traditionnels et le développement d'un esprit critique quant aux informations médicales sur le net.
